# Reply

**DOI:** 10.1111/jch.14415

**Published:** 2021-12-29

**Authors:** Yahui Liu, Chuanyu Gao

**Affiliations:** ^1^ Department of Cardiology Zhengzhou University People's Hospital Henan Provincial People's Hospital Zhengzhou China; ^2^ Henan Provincial Key Laboratory for Control of Coronary Heart Disease Zhengzhou University Central China Fuwai Hospital Zhengzhou China

We would like to thank Dr. Donatella Schiavone and colleagues for their interest and comments regarding our findings. We gladly take the opportunity to reply to their letter and add our concerns and viewpoints.

Renal denervation (RDN) is an emerging research field that has attracted much attention recently but has been controversial. The dispute focuses on whether RDN is effective and usable in treating patients with hypertension and resistant hypertension. It also questions whether the existing RDN from the intima of the renal artery (intima‐RDN) can reduce blood pressure and, subsequently, long‐term cardiovascular events.

Evidently, intimal injury is the initiating factor of atherosclerosis. Data from our team's previous studies suggest that intima‐RDN may induce or trigger renal artery injury or stenosis.^1^, ^2^, ^3^, ^4^ Therefore, similar to the opinion of Dr. Donatella Schiavone and colleagues, even if intima‐RDN can control blood pressure, it is not worth promoting.

In 2012, a rare opportunity for our team to implement RDN from the adventitia of the renal artery (adventitia‐RDN) in a patient with resistant hypertension produced surprising results.^5^ To date, the patient's blood pressure remains well controlled, and he has recovered well from preoperative stroke, cerebral hemorrhage, and neurological dysfunction. This result has aroused our great interest in exploring the adventitia‐RDN. Therefore, we designed a clinical trial on the treatment of resistant hypertension with adventitia‐RDN. However, after approval by our hospital ethics committee, the progress of patient enrollment was slow‐moving. We considered attributing this to the invasiveness of the surgical process, which may be traumatic for most patients who only take antihypertensive drugs. Thus, we revised our inclusion criteria to include patients with unilateral aldosterone‐producing adenoma (APA) complicated with resistant hypertension who needed laparoscopic surgery, considering that adventitia‐RDN is laparoscopic‐assisted. We obtained re‐approval from the ethics committee of our hospital afterward. All patients signed the informed consent and were enrolled in the clinical trial for about 6 years. Although the sample size was small, the feasibility, safety, methodology, and preliminary efficacy of adventitia‐RDN were preliminarily discussed. Surprisingly, we obtained positive results in lowering blood pressure and reported it in *The Journal of Clinical Hypertension*.^6^, ^7^

Although the antihypertensive mechanism of adventitia‐RDN remains unclear, several basic studies from our team and others’ have preliminarily explored the feasibility and effectiveness of adventitia‐RDN.^1^, ^2^, ^3^, ^4^ According to investigations, the mechanisms to consider may involve: (1) The severance of the sympathetic nerve distributed around the renal artery in the process of blunt separation of the renal artery and the exposure of its adventitia; (2) The removal of a part of the renal peripheral adipose tissue, thus reducing its influence in secreting unknown substances, secreted by adipocytes, on the sympathetic nerves; and (3) The reduction in sympathetic neurotransmitter release in the renal artery as adventitia‐RDN is more conducive than intima‐RDN, as shown by a study, which illustrated the renal sympathetic nerve being mainly distributed in the adventitia of the renal artery.^8^

Regarding the inclusion criteria, we complied with the American Heart Association definition of resistant hypertension. We checked and reanalyzed the research data by SPSS 22.0 software; the mean number of drugs ± SD (3.57 ± 0.69 and 3.39 ± 0.50 in the adventitia‐RDN and control group, respectively, shown in Table 3) was correct.^7^ According to the data mentioned above, the calculated 95% confidence intervals (CI) were 3.30–3.84 and 3.20–3.59, different from the 2.19–4.95 and 2.39–4.39 calculations by Dr. Donatella Schiavone and colleagues Furthermore, it needs to be explained that 95% CI is simply means that the probability of including a true mean is 95%, not that the probability that the true mean falls within the 95% CI is 95%.^9^ Therefore, according to the range of 95% CI, it may be inappropriate to conclude that there are patients taking less than three types of antihypertensive drugs in our study.

We have previously reported the duration of hypertension in the two groups of patients in earlier literature,^6^ and given that this is the same clinical study, we did not go over those details in the results of this 3‐year follow‐up paper.^7^ Due to the long follow‐up time and the large variability of patients taking antihypertensive drugs during the follow‐up period, we did not use the defined daily dose. Instead, but used the types of antihypertensive drugs to evaluate the changes in patients taking antihypertensive drugs before and after operation. It should also be noted that we did not rely solely on the difference of antihypertensive drug‐use to assess postoperative efficacy, but a combination of changes in blood pressure, antihypertensive drug‐use, and postoperative biochemical indicators (such as serum potassium and aldosterone‐to‐renin ratio values) for overall assessment.

The inclusion criteria of our study were different from those of the Primary Aldosteronism Surgical Outcome (PASO) study.^10^ The PASO study was aimed at unilateral primary aldosteronism patients, while our study was aimed at unilateral APA patients with resistant hypertension. Therefore, to evaluate the clinical and biochemical results, we used the previously described definition of “cured, improved, and failed,”^6^ which is different from the definition of “complete, partial, and absent success of clinical and biochemical outcomes” in the PASO study. In a previous paper, we have reported a 96.4% biochemical cure rate of patients a year after surgery,^6^ which corresponds to “over 95%,” as described by Dr. Donatella Schiavone and colleagues

Although the pathogenesis of hypertension remains ambiguous, the effect of sympathetic nerve activity on blood pressure is recognized. Sympathetic hyperactivity in patients with primary aldosteronism is controversial but not completely denied. A previous study has provided strong evidence for the association between excessive aldosterone secretion and sympathetic hyperactivity.^11^ According to the PASO standard, surgical treatment can achieve clinical success in only 37% of primary aldosteronism patients with hypertension,^10^ showing that a considerable number of primary aldosteronism patients still have hypertension after surgical treatment. This can be attributed to primary hypertension, severe heart and kidney damage, old age, metabolic disorders, and other factors. Our study shows that there may be activation of the sympathetic nervous system in patients with APA combined with resistant hypertension. However, further research is needed to explore the complex mechanism.

We share the view of Dr. Donatella Schiavone and colleagues that there is no question that unilateral laparoscopic adrenalectomy remains the gold standard for the treatment of patients with adrenal venous sampling‐proven unilateral APA. The ultimate goal of our study is not to challenge or even modify this gold standard, but to explore the feasibility, safety, and effectiveness of adventitia‐RDN in the treatment of hypertensive patients following numerous animal studies.

Thus far, many problems still puzzle us: (1) Do APA patients necessarily need RDN? (2) Is unilateral RDN effective? (3) Because the adventitia‐RDN operation process is complex, does it pose more damage to patients? (4) Even if additional adventitia‐RDN can be performed, will there be APA recurrence? What is the possible mechanism of recurrence? (5) Does implementation in the left or right renal artery of RDN affect the efficacy? (6) Can the antihypertensive efficacy after RDN be predicted by relevant indicators before operation? Further research is needed to solve the above series of questions.

We sincerely thank Dr. Donatella Schiavone and colleagues for their comments and the editor for the opportunity to conduct academic exchanges. This also allowed us to gain new insights into our study. We hope for more opportunities in the future to further discuss with Dr. Donatella Schiavone and colleagues topics under the field of hypertension.

## CONFLICT OF INTEREST

The authors have no competing interests.

## FUNDING

None.
